# Managing multiple, geographically-separated identities, and its effect on employee retention

**DOI:** 10.3389/fpsyg.2023.1189823

**Published:** 2023-08-31

**Authors:** Kerrie L. Unsworth, Ami N. Seivwright

**Affiliations:** ^1^Workplace Behaviour Research Centre, Leeds University Business School, University of Leeds, Leeds, United Kingdom; ^2^Institute for Social Change, College of Arts, Law and Education, University of Tasmania, Hobart, TAS, Australia

**Keywords:** multiple identities, identity management, employee retention, flexible work arrangements, fly-in fly-out (FIFO)

## Abstract

Extant literature posits that an individual manages their multiple identities by integrating or separating them to varying degrees. We posit that, rather than managing a single set of identities, an individual may engage different identity structures in different contexts. We use the fly-in, fly-out work context, whereby an employee’s home and work are substantially geographically separated, to explore whether different identity structures exist, strategies for managing them, and their effect on employee retention intentions. Analysis of qualitative data from 29 participants collected across three work sites revealed three main strategies that employees adopt to cope with having multiple identity structures: aligning identities; making work identity dominant; and creating a new identity around the working arrangement and discarding all other identities. These strategies interact with the employee’s actual identity structure to influence retention intentions. Implications for retaining employees in such working arrangements are discussed.

## 1. Introduction

Organizational research has conceptualized the management of the transition between work and home roles as an everyday task because, typically, the boundaries between work and home are crossed on an everyday basis (Ashforth et al., 2000; Rothbard et al., 2005). This research is often focused on the increasing permeability of role boundaries due to factors such as the encroachment of work into home via technology, alternative working arrangements such as teleworking and virtual teams and, more recently, the COVID-19 pandemic (Olson-Buchanan and Boswell, 2006; Sonnentag et al., 2010; Ashforth, 2020; Rudolph et al., 2021). However, there are working arrangements where the opposite situation exists - where boundaries between work and home are inherently distinct because the nature of the work sees the employee geographically separated from their home for long durations of time.

Fly-in, fly-out (FIFO) is one such arrangement. FIFO, also known as long-distance commuting, drive-in, drive-out (DIDO) or bus-in, bus-out, can be defined as “all employment in which the work is so isolated from the workers’ homes that food and lodging accommodations are provided for them at the work site, and schedules are established whereby employees spend a fixed number of days working at the site, followed by a fixed number of days at home” (Shrimpton and Storey, 1991, p. 27). These working arrangements are particularly common in the minerals and energy sector globally (Eilmsteiner-Saxinger, 2011; Misan and Rudnik, 2015; Donatelli et al., 2017; Paredes et al., 2018).

The FIFO working arrangement purports many benefits to workers, such as career advancement, high remuneration, increased choice of residential location, and extended recreation time (Misan and Rudnik, 2015). However, it also has its costs. Of particular concern to researchers and practitioners are the mental health impacts of FIFO on workers and their families, with studies finding high suicide rates, high divorce rates, and increased emotional and behavioral difficulties among children (Lester et al., 2016; Gardner et al., 2018). Partners of FIFO workers experience a range of impacts, including sleep disruptions, loneliness, and increased home duties (Asare et al., 2023), while the FIFO worker themselves report high psychological distress, isolation, and challenges regarding the transition from home to work and vice versa (Parker et al., 2018). These challenges often lead FIFO workers and their partners to liken their situation to “living two lives” (Parkes et al., 2005; Misan and Rudnik, 2015; Gardner et al., 2018). This may be further exacerbated by pandemic-induced changes to work, such that work travel is occurring less often (Caputo et al., 2021). This may mean that the home/work boundaries of the family and friends of FIFO workers are becoming more blurred (e.g., through work from home and less work travel), further emphasizing the contrast between FIFO and ‘normal’ work and the FIFO employees’ sense of isolation. Additionally, the mid-pandemic changes to the FIFO working arrangement, involving longer periods away from home and greater social isolation while on site to reduce infection risk had negative impacts on FIFO workers’ mental health which are predicted to have lasting ramifications on worker wellbeing and the tenability of the working arrangement for individuals (Gilbert et al., 2023).

In light of the challenges faced by FIFO workers, it is perhaps unsurprising that high turnover rates have been observed (Beach et al., 2003) and that some workers undertake FIFO work with a defined timeframe in mind before they prioritize other life such as having children (Misan and Rudnik, 2015). However, many also report staying longer than intended due to the “golden handcuffs” of higher remuneration than is attainable with more traditional working arrangements (Gardner et al., 2018). Therefore, there is evidence that FIFO workers experience ‘push’ factors that reduce retention intentions (Parker et al., 2018) while, simultaneously, factors within the job (mainly remuneration) inhibit the ‘pull’ of other employment opportunities. Given the high attrition and factors both drawing workers to and from FIFO arrangements, which are often centered on the challenges that FIFO poses to different roles that employees play in their lives, we posit that employee identity is a key missing link in our understanding of how employees navigate FIFO work.

Accordingly, this study seeks to explore the relationship between employees’ identities, the management of these identities, and retention intentions. We posit that the FIFO working arrangement is an ideal context in which to inductively examine the identity-retention relationship due to what Fruhen et al. (2023) describe as the “simultaneous fracturing and blending of personal and work lives” (p. 177) that characterizes FIFO, which, as we elaborate below, limits the extent to which employees can utilize established strategies to manage tensions between the different roles they play in life.

## 2. Theoretical development

As noted above, our research aim is to investigate, inductively, how employees manage complex interrelationships between identities and the subsequent effect on retention intentions. Social identity theory states that, in order to make sense of the social environment, individuals categorize themselves and others into social groups based on characteristics (e.g., age, membership of an organization or club) (Ashforth and Mael, 1989). The social identities that an individual holds then shape their behavior to align with their self-concept (Stets and Burke, 2000). In the context of organizational behavior, many studies have examined the role of social identity in building an employee’s emotional attachment to the organization (Van Dick et al., 2006) which can lead employees to remain in a project or organization (e.g., Haslam and Ellemers, 2005). Other retention research has examined an employee’s “other” identities such as their home identity (Wayne et al., 2006), their international-employee identity (Kraimer et al., 2012), or their age, ethnic, or sexual identity (Madera et al., 2012; King et al., 2017). However, to our knowledge, none of these bodies of work considers how an employee’s multiple social identities and strategies for managing these multiple identities relate to their retention.

The work that does examine multiple identities generally suggests that a person holds one set of identities (e.g., employee, mother, leader, partner) and that they manage these by either integrating them (e.g., I am an employee who is a mother) or separating them (e.g., I am an employee at work, and a mother at home) to various degrees (e.g., Brewer and Gardner, 1996; Ashforth, 2000; Nippert-Eng, 2008; Ramarajan and Reid, 2013; Ramarajan, 2014). Indeed, COVID-19 appeared to act as an identity shock that led employees to re-evaluate the relationship between these identities (Hennekam et al., 2021). Upon examining this body of research closely, it becomes apparent that there are two areas worthy of finer investigation. The first is that the identity structures (that is, the set/s of identities) are assumed to be relatively simple. Given the groundbreaking nature of this early work, this simplicity is understandable. Yet we know from experimental social psychology that identities, as long-term goals (Unsworth et al., 2014), hold both informational and motivational content (Kruglanski et al., 2002).

Roccas and Brewer (2002) theorize that if an individual perceives a high degree of overlap between the multiple ingroups to which they belong, then their identity structure will be simple. Roccas and Brewer (2002) propose further that, as simpler identity structures are easier to manage, individuals will adopt strategies to minimize the perceived differences between the different ingroups to which they belong. However, the inescapable differences between the groups to which one belongs often prohibit this simplification. We believe that identity structures also apply at the individual identity level, where instead of being defined by the social groups one identifies with and the perceived relationships between these groups, these individual identity structures comprise of the various roles one plays, such as parent, employee and friend, and the contextual relationships between them. We posit that integration or separation of a single set of identities does not always capture how identities are being managed. For example, does a CEO ‘separate’ their friend identity while they are presenting to the Board, or is their friend identity absent in this context? While a seemingly small difference on the surface, the difference between separating (‘I am always a friend but at work I prioritize my role as CEO’) and suppressing (‘At work I am only a CEO’) identities has implications for how identities within an identity structure relate to each other and how these relationships are managed.

Certain contexts amplify the potential difference between identity structures. For example, using the context of this study, a FIFO worker cannot tuck their child into bed or help with the dishes during their (typically 1–4 week) stint at work and, conversely, cannot work while at home. Therefore, we suggest that identities will differ not only in their presence or absence in different contexts, but also in their perceived importance. In addition, we suggest that the relationship between identities in each context may be different; for example, while at home being a leader may conflict with being a wife (if say, the husband does not like the wife working) but at work these two identities, while still salient, may not be perceived to be in conflict.

The second area worthy of finer investigation is the relationship between the identity structure and the identity management strategy. As can be seen above, there is some research which seems to focus more on the identity structures (e.g., Ashforth, 2000; Ashforth et al., 2000) and other research which seems to focus more on the strategies used to manage the different identity structures (e.g., Nippert-Eng, 1996; Kreiner et al., 2006, 2009; Ramarajan and Reid, 2013). We believe that as we start to understand more about how a person deals with multiple identities, we need to clearly separate these two constructs and examine the interplay between them.

Finally, this study explores the relationship between one’s identity structures and concomitant identity management strategy, and their retention intentions. Recent literature has found that the decision to leave is often a result of seeking identity congruence across life domains (Rothausen et al., 2017). Accordingly, this suggests that the way in which employees manage their different identity structures will affect their retention intentions, yet we know very little about the nature of this relationship. We posit that geographically separated work is an apt context in which to explore employees’ identity structures and their associated identity management strategy as the employee cannot simply separate or, alternatively, integrate their identities (Ashforth et al., 2000).

## 3. Methods

Given the exploratory nature of this research, and the complex, intertwined nature of multiple identities, inductive qualitative methods were judged to be the best approach to examine the research questions. We conducted two studies: a pilot study to ground the questions and provide a comparison for checking theoretical saturation, and the main study which provided most of the data.

### 3.1. Sample

For our pilot study, a snowball sample was used (Biernacki and Waldorf, 1981) consisting of six males working on mine sites located at least 1,000 km from the nearest city. A convenience sampling approach was used, in which a FIFO worker known to one of the authors asked colleagues if they would like to participate in an interview about their experiences working FIFO. Those who indicated interest were given the author’s contact details and then an interview was organized at an agreeable time and location. Prior to the interview commencing, participants were given a Participant Information Form, asked if they had any questions, and then asked for both written and verbal consent. All participants were on a roster that consisted of two weeks on site (7 days of 12-h day shifts followed by 7 days of 12-h night shifts), and one week at home. Two of the participants were over 30 and married with children. Three were single and aged between 23 and 27, and one was 20 years old and engaged. Two were auto-electricians, three were diesel fitters and one was a driller.

A main sample was then interviewed from a multinational mining company. One author traveled to three mine sites, all approximately 1,000 kms from the capital city, and interviewed a total of 23 employees (*N* = 8 at site 1; *N* = 8 at site 2; *N* = 7 at site 3). Participants from the main sample were referred to the research by the employing organization. A Participant Information Form was provided, participants were asked if they had any questions, and were then asked if they wished to proceed. Those who were happy to proceed signed a consent form and consent was verbally confirmed prior to the commencement of the interview. Fifteen of the main sample participants were male, eight were female; 14 were married or in relationships and nine were single; and participants ranged in age from 22 to over 51 years old, with a mean age of 36 years. See Table 1 for sample information.

**Table 1:** Main sample demographics.

Participant	Sex	Age	Job category	Marital status	Children	Tenure with organization	Roster (days on site/days at home)
1	M	31–40	Site Management	Single	No	9 months	8/6
2	M	31–40	Geology	Married	Yes	8 years	8/6
3	F	26–30	Occupational Health and Safety	In a Relationship	No	1 year	8/6
4	F	26–30	Occupational Health and Safety	In a Relationship	No	1 year	4/3
5	F	26–30	Environmental Management	Single	No	5.5 years	8/6
6	M	51+	Site Management	Married	Yes (adult)	4 years	7/7
7	F	51+	Site Management	*De Facto*	Yes (adult)	3 months	8/6
8	M	22–25	Engineering	Single	No	3 months	12/2
9	F	31–40	Occupational Health and Safety	Married	No	3 years	7/7
10	M	22–25	Professional Services	Single	No	1 year	4/3
11	M	31–40	Professional Services	In a Relationship	No	6 months	Alternating 8/6 and 4/3
12	M	51+	Site Management	Married	Yes (adult)	12 years	8/6
13	M	26–30	Engineering	In a Relationship	No	4 years	8/6
14	M	31–40	Geology	Married	No	1.5 years	8/6
15	M	26–30	Environmental Management	Single	No	1 month	8/6
16	F	51+	Professional Services	Single	Yes (adult)	1 year	8/6
17	M	26–30	Geology	*De Facto*	No	3 years	8/6
18	F	41–50	Geology	Single	No	3.5 years	Alternating 8/3 and 8/9
19	M	31–40	Site Management	*De Facto*	Yes	6 years	8/6
20	M	31–40	Site Management	In a Relationship	Yes	6.5 years	7/7
21	M	41–50	Professional Services	Married	Yes	6 years	Alternating 8/6 and 4/3
22	M	22–25	Geology	In a Relationship	No	10 months	7/7
23	F	51+	Professional Services	Single	Yes (adult)	1 month	Alternating 8/6 and 4/3

All except one participant in the main sample worked a roster consisting of equal or almost equal amounts of time spent on site and at home. All participants worked only day shifts averaging 13 h in length. Participants worked in departments such as occupational health and safety, engineering, and metallurgy; additionally, there were some clerical staff and some technical staff in supervisory roles.

### 3.2. Interview design and data collection procedures

#### 3.2.1. Pilot testing

To capture their identities, participants in the pilot study were first provided with the 20 statements test (Kuhn and Partland, 1954), asking the participant to write out 20 short statements answering the question “who are you”. Participants were asked to first consider “who they were at work”, then “who they were at home”. A semi-structured interview was then conducted using the critical incidents technique (Flanagan, 1954). The rest of the interview schedule was developed around the extant literature on retention, identity theory and FIFO. To capture retention intentions, participants were asked how long they intended to stay in their current job. In addition, questions were asked about sources of satisfaction and dissatisfaction, the participant’s degree of organization and societal fit and the importance they place on it, and the process of adjusting between being at home and being on site.

#### 3.2.2. Main sample

The interview schedule for the main sample was adjusted according to observations from the data gathered from the pilot study, as well as difficulties noted during the administration of the interviews to the participants of the initial study. Participants found it difficult to respond to the 20 statements test (Kuhn and Partland, 1954) so this was replaced with questions based upon goal hierarchy theory (Unsworth et al., 2014). Possible identities were identified from the initial study and the literature and included both roles such as friend, partner, parent, and descriptors such as reliable, friendly and good employee. As this study was part of a broader project, the goal hierarchy template also included, in addition to identities, more concrete goals (i.e., project goals and task goals), but these were not used for this study. The fully-populated goal hierarchy was copied onto both sides of one sheet of paper. Participants began with one side and were asked to think about themselves when they were at work. First, they reviewed all identities, project goals, and task goals and crossed out any that were not relevant to them when they were at work. Second, they were asked to tick those that were of particular importance to them at work and to draw relationships between them. The participants then turned the page over and began the process again, but this time considering themselves when they were at home.

To further triangulate our understanding of any differences between home and work identities, we added an explicit question at the end of the interview asking the participants if they felt that they adopted different identities on site versus at home. As such, we used both atomistic (content differences) and molecular (perceived differences) approaches (Edwards et al., 2006) to studying the relationship between the home and work identities. Finally, in response to the role conflict reported by the initial sample, a new critical incident was added to the interview schedule asking the participants to reflect on whether they had felt conflict between the different roles they play in their life.

### 3.3. Analytical strategy

As the relationship between social identity theory and employee retention has not been explored thoroughly, especially in relation to FIFO workers, we used an inductive approach to analysis. In particular, we followed a process of coding, identifying concepts, and categorizing to gather a collection of explanations (Strauss, 1987). Each interview transcript of both the pilot and the main samples was subject to intensive open coding to identify the themes that were present across the data set. A reliability check of the open coding was performed by a colleague not involved in the study to ensure accuracy before further analysis was conducted – there was over 85% agreement and all discrepancies were discussed before finalizing the coding. Axial coding was conducted on the main sample data to make connections between categories and themes and to construct emerging theory (Strauss and Corbin, 1990). Throughout the analysis, negative examples were actively sought to disprove these emerging conclusions and, when identified, the theory was built upon to incorporate those findings.

## 4. Results

Our research presumes that FIFO workers could indeed have multiple identities and that the structures of those identities, in terms of their presence and salience, could vary across contexts. As can be seen in Table 2, this was clearly the case in our sample.

**Table 2:** Summary of results.

Strategy	Similarity across identities	Retention outcome	Participants
Aligning work and home	High similarity	Strong retention intentions	12, 14, 16, 21
	Moderate similarity	Moderate retention intentions	2, 7, 8, 18, 19
Making work identity dominant	High similarity	Strong retention intentions but “pull” vulnerabilities	5, 6, 10, 15
	Fewer work identities in home	Moderate retention intentions and “pull” vulnerabilities	3, 4, 11, 13, 17, 22
Creating new FIFO identity		Strong retention intentions	1, 9, 23

Most participants had different identities both on site and at home and only a very few felt that the identities held the same level of importance to them across both work and home. Thus, most participants indicated on their goal hierarchy sheets that their identities depended to some extent on where they were located at the time. Our first finding, therefore, is that many FIFO workers have different identity structures in the different contexts of their life. Given that we asked people to consider both contexts one after the other we would expect similarities rather than differences across the structures due to common-method bias and the psychological desire for consistency (see e.g., Cialdini and Goldstein, 2004); thus, we believe this is a strong finding.

### 4.1. Managing different identity structures

In light of the finding that FIFO workers often adopt different identity structures in different contexts, we then examined how they dealt with the multiple identities and the effect that this had on their retention intentions. We found that the identity strategies did not always align with the identity structures, so we consider them separately and examine the interplay between them.

We identified three prominent identity management strategies that all except one participant discussed: (1) Aligning identities; (2) Making work identity dominant; and (3) Creating a new FIFO identity. Three very distinct groups of participants emerged, yet these groups were distinguished only by the manner in which they managed their multiple identities. Diversity of demographic characteristics in terms of gender, age, job role, whether or not they had children, how long they had been in their current role and how long they had been working FIFO is seen across all three of the identity strategies. The three different management strategies were associated with substantially different views on retention. Further, within the strategies there were differences in the effect on retention based on the identity structures. We categorized employees’ retention intentions as weak if they expressed that they were actively seeking jobs with different organizations at the time of interview, moderate if they indicated intention to stay in their current organization 2–3 years and/or stated that they would seriously consider other job offers if they were approached, and strong if they indicated an intention to stay five or more years.

#### 4.1.1. Identity strategy 1: aligning identities

The first strategy we identified was attempting to have parallel identity structures across both home and work. In response to the specific interview question about their “work” self and their “home” self, these participants believed that they had the same identities both at home and at work. We described these participants as using an “aligning” strategy to negotiating their multiple identities.

Although they considered themselves to have similar identities across both work and home, it is important to note that many participants who used this strategy still saw themselves as having two separate lives and not one amalgamated self-concept. Indeed, participants who aligned their identities often dealt with any negative feelings they may have had toward their working arrangement by focusing on the benefit that FIFO offered both sides of their lives. For instance, Participant 16 said, “I can indulge myself when I’m at home and I can work hard when I’m here and I do not have to feel guilty,” while Participant 12 explained that “..the time you spend at home is better quality time because you can take your kids to school and pick them up, you know, you can take them to sporting events afterwards.”

When examining the identity structures of participants using an aligned strategy, we found that participants did often display similar home and work goal hierarchies. However, while there were similarities, they were rarely identical, which is what would be expected if the aligning strategy was working completely (i.e., if a person was truly able to align their identities in both home and work contexts). The level of similarity of identities across work and home identity structures ranged from 55 to 100%. Interestingly, half of the participants who discussed using an aligning strategy had less than 75% similarity in their identity structures; indicating that the strategy of wanting to be the same person at home and at work is not being achieved for a significant portion of our sample.

At a general level, all participants who said that they aligned their identities reported that they intended to remain within the organization. However, as can be seen in Table 2, there appeared to be a relationship between the similarity of the identity structures and the strength of their retention intentions. Those participants with complete similarity reported strong retention intentions, while those with partial similarity reported only moderate retention intentions. There was only one exception and this person (who had complete overlap) had changed jobs regularly throughout their career. Thus, what we considered a “moderate-strong” retention intention may well be a strong retention intention given his background. Thus, we believe that the data support our conclusion that an aligning strategy with complete overlap in the identity structure leads to strong retention intentions while an aligning strategy with moderate overlap is related to only moderate retention intentions. Table 3 provides examples of participants using the aligning strategy, including their identities and overlap on the goal hierarchies they completed, and quotes from their interviews.

**Table 3:** Aligning strategy.

Participant	Identities (home)	Identities (work)	Number of overlapping identities	Evidence of coping strategy	Retention intentions	Evidence of retention intentions
2	Friend, Partner, Parent, Reliable	Friend, Partner, Parent, Career-oriented, Part of the team on site, Reliable, Good Employee	4/7	They [my career goals] are important, but you know, they do not come before my family. I’m not going to ah, neglect my family to sort of get to the process manager position.. So, I’d like to get there, but yeah, I’m not too keen on taking time away from the family..at this stage where I am at the moment, I can go home and just forget about work, and I like that.	Moderate	Again, this job I could do til I retire. Um, yeah at [Organization]? Oh, who’s to say? Again, if something were to come up, or you know, if there was an attractive offer, I’d have a look at it, but as for now, I’m happy.
12	Friend, Partner*, Parent*, Career-oriented, Part of the team on site*, Reliable*, Good employee	Friend, Partner*, Parent*, Career-oriented, Part of the team on site*, Reliable*, Good employee	7/7	When I got to that 5 year period I was able to say “well, everything’s good at home, I do not really need to come home and sort wayward kids out” so yeah, that was ah, that was about it, that’s how I set goals the time you spend at home is better quality time because you can take your kids to school and pick up	Strong	..it was a two year plan and I said I’d give it two years, no problem. Then I looked at the five years because that’s when my kids would be teenagers, and once that happened, now I’d be quite happy just to float on, and quite honestly I cannot see that changing
21	Friend, Partner*, Parent*, Career-oriented*, Part of the team on site*, Reliable*, Good employee	Friend, Partner*, Parent*, Career-oriented*, Part of the team on site*, Reliable*, Good employee	7/7	My own experience is that fly-in, fly-out is a much more rewarding lifestyle than a residential lifestyle would be and I certainly feel that when I’m at home I can give quality time and I can enjoy quality time with my family and that’s important to me	Strong	My goals are to continue to stay with [Organization] and hopefully continue to build a management career with [Organization], essentially, and I will only do it as a fly-in, fly-out

*Denotes self-reported salience.

Our interviews also revealed different factors affecting retention based on particular identity strategies and structures. Many participants who aligned their identities stated that knowing what to expect from the FIFO arrangement helped them with accepting the negatives. Participant 14 said, “We both knew that when I was coming over for the job, that [being away] was what was going to get involved and that’s what…we knew what was coming so some days it falls in your favor and other days it does not” while Participant 16 said, “…while I had not worked fly-in, fly-out, I had worked in a local mining industry, heavy industry. So I was comfortable with that sort of culture.” Thus, it appears that knowledge of and expectations management around FIFO may facilitate an effective alignment strategy.

In summary, therefore, many participants chose an aligning strategy where they tried to be the same person at home and at work, while acknowledging that these two different sides to themselves existed. The extent to which their alignment strategy was successful appeared to influence retention intentions.

#### 4.1.2. Identity strategy 2: dominant work identity

The second strategy for dealing with multiple identities was based around making the work identity dominant. Participants who used this strategy focused on the unparalleled benefit to their career. For example, Participant 3 stated that she appreciated being able to work “fly-in, fly-out on a mine site and still pursue the medical [career] interests” she has, and while Participant 10 still felt he “had a lot to learn” and “missed the team” at his previous job, the FIFO opportunity “opened up so much more for [him] here.”

Participants who created a dominant work identity seemed to suppress thoughts of other aspects of themselves; e.g., when asked about future goals outside of work, one participant answered with “I have not really thought about it, I’m more career-oriented at the moment” (Participant 13). Other participants, when discussing what they missed out on at home, acknowledged that they missed out on “some stuff [but] that’s just part of it” (Participants 5 and 6) or “it’s not really a big deal” (Participant 10). The dominant work identity influenced their experience of FIFO as every decision, from choosing to begin their FIFO career to choosing to stay in their current role, was analyzed almost purely from a career-based perspective. For example, Participant 6 stated that when he made the decision to take the job, that “…well it was just a career choice really,” and Participants 10, 11 and 13 all said that it was the career progression opportunities that led them to enter and stay in their current job.

A subset of this category focused specifically on the monetary rewards of the work and the potential benefit that financial security offered their other identities. For instance, Participant 17 stated that “while it’s just me and my partner, before we have kids, might as well try to earn as much; the earning potential is at a great peak right now and that’s definitely a great incentive and motivator to keep me out here.”

When examining the identity structures of participants who used a dominant work strategy we found that many had high levels of similarity across the home and work contexts. All of them included work-based identities such as being career-oriented, part of the team on-site and a good employee in their “at home” identity structure. For these participants, the differences in identity structure occurred through the lack of home-based identities in the “at work” identity structure. For example, being a partner was simply not included in the “at work” identity structure for three participants, even though they had an identity as a partner while at home.

In general, participants who used a dominant work strategy had moderate to strong retention intentions. The process of managing the identities again appeared to interact with the identity structure in relating to retention. In this case, what was important was the degree to which the work identities were salient when the participant was considering themselves at home. As can be seen in Table 2, when a work-dominant strategy was used, and the participant included all their work-related identities in their home identity structure, then there were strong retention intentions (participants 5, 10, 15). When that strategy was used but only some work-related identities were included in their home identity structure then the retention intentions were only moderate (participants 3, 11, 13, 17, 22). Two participants did not fit this finding – both did not have work-related identities in their home identity structure but had strong retention intentions. However, one was close to retirement and the other was working on an internship. Career stage may therefore be a moderating factor in the relationship between a work-dominant identity management strategy and retention intentions. Generally, however, our results suggest that a strategy of making the work identity dominant is associated with strong retention intentions when work-based identities are also important at home, but only moderate retention intentions when not all work identities are important at home. Table 4 provides examples of participants using the dominant work identity strategy.

**Table 4:** Dominant work identity strategy.

Participant	Identities (home)	Identities (work)	No. overlapping identities	Evidence of coping strategy	Retention intentions	Evidence of retention intentions
3	Friend*, Partner*, Reliable*, Career-oriented, Part of the team on site, Good employee, Gym Junkie	Friend, Partner*, Career-oriented*, Part of the team on site*, Reliable*, Good employee*, Gym junkie	7/8	I’m doing some further study, some further paramedical science study, and also I’m doing my masters in occupational health and occupational science. And I’m also studying and finishing a geology degree. So yeah, career-wise I’d like to move into management	Moderate	So I was looking two years plus, and I still am. I look to see what’s going, but I never actively apply. I just like to see what’s going on in the market and what sort of money people are getting for the job I do.
5	Friend, Career-oriented, Part of the team on site, Reliable*, Good employee*	Friend, Career-oriented, Part of the team on site, Reliable*, Good employee*	5/5	Future goals. Ah, long term would probably be moving up to environmental manager, at some point.. so I have a personal development plan, that I think is very good that we do, so that has all the training and the things that I need to do to get to that next position So, umm, I do not really have that problem [role conflict].	Strong	Probably a lot more years (laughs). 15 or something. I dunno, we’ll see
17	Friend*, Partner*, Career-oriented, Reliable*, Good employee	Friend, Career-oriented, Part of the team on site*, Reliable*, Good employee*	3/6	Rather than having a job, I have a career and with that the chance to move up and progress, rather than having a dead-end job or a job that did not have any prospects in terms of career advancement	Moderate	While I’m still happy and still learning, and the job’s not mundane, not boring, not repetitive, I’m quite happy to stay

*Denotes self-reported salience.

Finally, although participants who adopted a dominant work identity strategy reported an intention to stay in their current role, when asked if alternate job offers would influence them, they stated that if the conditions were comparable (for example: roster, accommodation, job role) that they would consider it.

In summary, some participants managed their multiple identities by making their work identity dominant. Participants who used this strategy were influenced by sacrifice factors when considering their retention intentions more than participants who used other strategies. Importantly, though, for these participants, it was the degree to which their work identities were salient and important while at home that affected their retention intentions.

#### 4.1.3. Identity strategy 3: FIFO identity construction

The final category of participants built an entirely new identity around the working arrangement. This can be seen in statements such as “I’ve built my life around FIFO” or “You just have to learn to fit in.” These participants identified themselves first and foremost as FIFO workers and molded their other identities around this. They felt that the culture of FIFO required the adoption of an entirely different identity. Indeed, we were told that FIFO “really” stands for “Fit-In-or-F***-Off.” This strategy required the abandoning of any identity that did not center on FIFO. For example, Participant 1 stated “Um, look, well because I do not have a social aspect, I’ve set up my life to fit this, so it’s [FIFO] not an impact [on my social life] at all.”

Unsurprisingly, these participants showed high similarities across their work and home identity structures. These participants also reported very strong retention intentions, regardless of the level of overlap between the identity structures. For example, Participant 23 stated that she would be in FIFO and her current role “for life.” Table 5 provides examples of these participants.

**Table 5:** FIFO as an identity strategy.

Participant	Identities (home)	Identities (work)	Number of overlapping identities	Evidence of coping strategy	Retention intentions	Evidence of retention intentions
1	Friend, Part of the team on site, Reliable, Good employee	Friend, Career-Oriented, Part of the team on site, Reliable, Good employee	4/5	..well because I do not have a social aspect, I’ve set up my life to fit this, so it’s not an impact [on my social life] at all if you do not make it [in FIFO], go away and do not come back because it is hard, and you have to have a different mind set	Strong	So, now it’s [my future goal] definitely to be successful at my job, to do a good job. Do it consistently, and build up a person to fill in my shoes and then to diversify in [Organization]
9	Friend*, Partner*, Part of the team on site, Reliable, Good employee	Friend, Partner*, Part of the team on site*, Reliable*, Good employee	5/5	We actually rent out our house and live on a boat when we are off site… we have built our life around FIFO	Strong	We could never go back to a 5 and 2 roster in Perth

*Denotes self-reported salience.

Interestingly, this was the strategy that was used by the fewest number of participants. It could be that the creation of a new identity and discarding “the other self” is too difficult for many people. However, we believe that such an extreme identity coping strategy for dealing with a working arrangement is an important finding both for understanding the FIFO working arrangement and developing the identity literature.

## 5. Discussion

This study explored how employees manage their multiple identities in the unique FIFO working arrangement and how that related to their retention intentions. While COVID-19 has tended to blur boundaries between work and home (Rudolph et al., 2021) with subsequent effects on identity (Ashforth, 2020; Hoff, 2021), the FIFO arrangement remains one where these contexts remain distinct, and as such it provides an extreme case from which new knowledge can be discovered. First, we found variation in the number of identities, the levels of importance of those identities, and the degree of overlap between identities employees held in the work and home contexts – it was not that employees reconstructed their home and work identities within a single structure [as per during the COVID-19 identity shock, Hennekam et al. (2021)], but that they constructed separate identity structures for each context, each housing variants of work and home identities. Second, there was complexity in the different identity management strategies that people used beyond the degree of separation versus integration identified in previous research. Finally, there was complexity in the interplay between the structures and the strategies and the effect that this had on retention intentions. Moreover, we found that the use of different identity management strategies related to the relative importance of other job factors. We discuss all these aspects of the results and how they relate to the broader literatures below.

Retention research to date has focused predominantly on factors affecting embeddedness, namely links, fit, and sacrifice (Felps et al., 2009; McEvoy and Henderson, 2012; Murphy et al., 2013) while turnover research has focused on push and pull factors (Griffeth et al., 2000; Lee et al., 2006). Our research has shown that the way in which people manage their different identity structures across their home and work lives affects which of these factors is important in a retention decision. Specifically, we found that people sought different types of identity fit - whether that be aligned across work and home, having their work identity dominant, or creating an entirely new identity around FIFO and discarding all others.

Identity management and the reconstruction or adoption of dual identities is not a new concept (Ramarajan, 2014). However, in our research we distinguished between identity structures (the set of identities that people had in each of the work and home contexts) and the process through which they managed these two structures. Work on dual identities has tended to focus on the structure in only one context (e.g., Brewer, 1999; Kreiner et al., 2006, 2009; Ramarajan and Reid, 2013) or a combined context, as in the case of working-from-home during COVID-19 (e.g., Hennekam et al., 2021). We looked at identity structures in two different contexts and found that most participants maintained two separate identity structures, they just differed in the extent to which these structures were similar to each other. Notably, even those who felt they were the same person at home and at work acknowledged their different “selves.” The only people who did not have distinct identity structures were those who created a FIFO identity. Furthermore, we found that for our FIFO employees, the emphasis was not on managing the integration of specific identities while at work, it was instead about managing the broader identity structures that emerged at work versus at home. Thus, we identified new ways in which people manage these different identity sets: aligning, making work identity dominant and creating a new FIFO identity.

What we find particularly interesting is the relationship between the identity management strategy, the identity structure and retention intentions. While it is not possible to determine causality, we posit some interpretations for why these findings emerged. First, we found that the aligning strategy was related to strong retention intentions when there was high similarity between the identity structures but only moderate retention intentions when there was moderate similarity between the identity structures. We suggest that the aligning strategy is essentially trying to create two different “lives” where one life helps the other. If these “lives” are completely in sync, then this spillover is more able to occur; if the “lives” are not in sync then the spillover is less likely to emerge, and the employee is less likely to get the benefits that they were hoping for. It is important to note that both external and individual contextual factors may interfere with an individual’s ability to align their home and work lives. For example, if working from home and increased childcaring responsibilities are blurring a partner’s home/work lines and increasing stress, this could impede the FIFO worker’s ability to frame FIFO as something that serves both of their “lives.” With regard to external factors, studies of FIFO during COVID-19 found increased psychological distress (Gilbert et al., 2023) and high rates of negative health behaviors such as smoking and drinking (Asare et al., 2022). Therefore, external factors may, for various reasons, make FIFO more (or less) difficult to balance.

Second, we found that the work-dominating strategy was less likely to lead to strong retention intentions if the work-based identities were not included in the home identity structure. This strategy is based on the premise that the person focuses on work in order to deal with the separation of identities; if they do not, or are not able to, focus on work while at home then the strategy is not working completely, and they may feel the negative effects of being away from home for so long (see Taylor and Simmonds, 2009). In other words, those people in our sample who did not include work-based identities in their home structure may be using a strategy that is not appropriate for them or may not be using the strategy effectively.

Finally, the idea that some FIFO workers adopt an entirely new identity as an identity coping strategy rather than merely self-categorizing already existent aspects of their self-concept is a very interesting finding. Participants using either of the first strategies may be finding the optimal balance between identity structures (see Kreiner et al., 2006) by viewing the FIFO arrangement from the perspective of their salient identities. Yet, this last category of employees is choosing not to strike a balance but instead to eliminate identities entirely and create a different one.

Overall, with regard to retention intentions, it appeared that it was the extent to which a person’s strategy was successful which was related to retention. In other words, not all factors are equally important for all FIFO employees. This is an important contribution to the literature as it identifies a clear moderation of factors. When work identities are considered most important then sacrifice work-factors such as remuneration and career progression opportunities are the key issues. Alternatively, when a person aligns their work and home identities then fit is vital. Finally, when a person created an entirely new identity then retention intentions were not even relevant; thus, it appears that the creation of a new FIFO identity is a factor related to retention in and of itself. This is important as identity management has not previously been examined closely in regard to retention (see, e.g., Maertz and Griffeth, 2004).

The impact of identity and identity coping strategy on retention intentions has implications for organizations attempting to retain a FIFO workforce. Results from this research indicate that one cannot infer an employee’s retention intentions from their demographic characteristics or family situation. Instead, it will be important for organizations to consider how employees are managing the different roles they play in their lives. Interestingly, network marketing organizations have been found to leverage work–family conflict into commitment from their workers by bringing “family” into work (Pratt and Rosa, 2003). Similarly, collective investiture socialization tactics have been shown to affect turnover (Allen, 2006). We suggest that these practices may be attempting to align the employees’ identity structures or promote the creation of an identity structure based around the job or organization. Organizations using the FIFO arrangement may increase retention by facilitating the management of different identity structures, such as arranging family site visits, ensuring access to reliable telecommunications infrastructure, or even simply acknowledging the challenges of being away from home (Misan and Rudnik, 2015; Albrecht and Anglim, 2018).

### 5.1. Limitations and future research

Given that the role of identity theory and employee retention is relatively unexplored in the literature, especially in the FIFO context, a qualitative study was an ideal method to begin examining the relationships. We were also fortunate that we were able to obtain a reasonable sample over multiple mine sites. However, several themes arose from this study that could benefit from longitudinal examination using both quantitative and qualitative methods. For instance, the impact that an employee’s identity management strategy has on their turnover behavior over time could be explored either with a longitudinal study or by seeking a sample with participants who have recently left their jobs. In addition, examining identity structures and whether the identity coping strategies identified in this study apply in different contexts will further develop our understanding of identity.

## 6. Conclusion

This study explored the relationship between the identity structures and identity management strategies of fly-in, fly-out workers, whose working arrangement sees their home and work contexts geographically separated for extended periods of time, and the impact of these structures and management strategies on employees’ retention intentions. We find that many employees do adopt different identity structures (sets of identities and their relative salience) in their home and work contexts, and identify three main strategies utilized by employees to manage these identity structures. These strategies were aligning work and home identities, making the work identity dominant, and adopting a new identity around fly-in, fly-out work. The extent to which these strategies worked, such that the identities employees identified as present and salient in home and work contexts ‘matched’ their identity management strategy, appears to be associated with the strength of retention intentions. The results suggest that employers could increase retention by considering the strategies employees are using to manage their multiple identities and introduce initiatives and/or infrastructure to support these strategies.

## Data availability statement

The datasets presented in this article are not readily available because the data generated from the manuscript are qualitative and too potentially identifiable for release. Requests to access the datasets should be directed to ami.seivwright@utas.edu.au.

## Ethics statement

The studies involving humans were approved by The University of Western Australia Ethics Committee. The studies were conducted in accordance with the local legislation and institutional requirements. The participants provided their written informed consent to participate in this study.

## Author contributions

KU and AS contributed to all components of the study (conceptualization, design, analysis, writing). KU led the writing and intellectual development of the paper. AS led the data collection and analysis. All authors contributed to the article and approved the submitted version.

## Conflict of interest

The authors declare that the research was conducted in the absence of any commercial or financial relationships that could be construed as a potential conflict of interest.

## Publisher’s note

All claims expressed in this article are solely those of the authors and do not necessarily represent those of their affiliated organizations, or those of the publisher, the editors and the reviewers. Any product that may be evaluated in this article, or claim that may be made by its manufacturer, is not guaranteed or endorsed by the publisher.
